# Gastrointestinal Parasitic Infections in *Macaca fascicularis* in Northeast Thailand: A One Health Perspective on Zoonotic Risks

**DOI:** 10.3390/ani15142112

**Published:** 2025-07-17

**Authors:** Teputid Kuasit, Manachai Yingklang, Penchom Janwan, Wanchai Maleewong, Weerachai Saijuntha, Siriporn Kuanamon, Tongjit Thanchomnang

**Affiliations:** 1 Faculty of Medicine, Mahasarakham University, Maha Sarakham 44000, Thailand; teputid.k@msu.ac.th (T.K.); weerachai.s@msu.ac.th (W.S.); siriporn.ku@msu.ac.th (S.K.); 2 Faculty of Public Health, Burapha University, Chonburi 20131, Thailand; manachai.yi@buu.ac.th; 3 Department of Medical Technology, School of Allied Health Sciences, Walailak University, Nakhon Si Thammarat 80160, Thailand; penchom.ja@wu.ac.th; 4 Department of Parasitology, Faculty of Medicine, Khon Kaen University, Khon Kaen 40002, Thailand; wanch_ma@kku.ac.th; 5 Mekong Health Science Research Institute, Khon Kaen University, Khon Kaen 40002, Thailand; 6 Biomedical Science Research Unit, Mahasarakham University, Maha Sarakham 44000, Thailand

**Keywords:** long-tailed macaque, gastrointestinal parasites, prevalence, One Health

## Abstract

Gastrointestinal (GI) parasitic infections in non-human primates are of growing concern due to their implications for both veterinary and public health. Long-tailed macaques (*Macaca fascicularis*), commonly found in peri-urban and temple environments in Southeast Asia, may act as reservoirs for zoonotic parasites, posing risks to humans and domestic animals. This study investigated the prevalence and species diversity of GI parasites in free-ranging macaques from four provinces in Northeast Thailand (Loei, Khon Kaen, Bueng Kan, and Sisaket). A cross-sectional study was conducted between April and May 2025. A total of 445 fecal samples were examined using two parasitological techniques: agar plate culture (APC) and the formalin–ethyl acetate concentration technique (FECT). The overall prevalence of parasitic infection was 86.5%, with *Strongyloides* sp. (65.2%) as the most prevalent helminth and *Balantioides coli*-like (29.5%) and *Entamoeba histolytica*-like (28.8%) as the predominant protozoa. Other parasites identified included helminths (*Trichuris* sp., *Ascaris* sp.) and protozoa (*Blastocystis* sp., *Iodamoeba bütschlii*, *Entamoeba coli*, and *Chilomastix mesnili*). Mixed infections were frequently observed, with both helminths and protozoa co-occurring in 37.3% of cases. The high infection rates and parasite diversity reflect substantial environmental contamination and sustained transmission cycles. These findings underscore the importance of integrated surveillance in wildlife populations and the need for One Health-based approaches to minimize zoonotic transmission risks at the human–animal–environment interface.

## Simple Summary

Long-tailed macaques in Thailand often live close to humans in places like temples and parks. This close contact can increase the risk of spreading diseases, including intestinal parasites that affect both animals and people. In this study, we collected fecal samples from macaques in four provinces of Northeast Thailand and tested them using two laboratory methods. We found that most macaques (86.5%) were infected with at least one type of parasite. The most common parasites were *Strongyloides* sp. (a type of worm) and *Balantioides coli*-like (a protozoan). Some macaques were simultaneously infected with multiple parasites, which may reflect environmental burden and potential transmission complexity. This highlights the importance of monitoring macaque health and managing human–wildlife interactions to reduce disease risks.

## 1. Introduction

Emerging infectious diseases, especially zoonoses, pose serious threats to global health. These diseases show the close connection between human, animal, and environmental health [1]. The One Health concept recognizes that the health of people is closely linked to the health of animals and shared ecosystems [2]. Among these zoonotic agents, gastrointestinal (GI) parasites are a persistent and often neglected concern. They are transmissible across species and pose public health concerns. Globally, more than 1.5 billion people are infected with GI parasites, particularly soil-transmitted helminths [3]. Many domestic animals, non-human primates, and wildlife also carry these GI parasites [4,5,6,7,8]. Additionally, parasitic eggs and/or cysts have been detected in soil, water, and other parts of the environment [9,10,11,12]. These findings suggest ongoing transmission cycles between animals, humans, and their surroundings.

In Thailand, GI parasites are still common, especially in rural and high-contact areas [13]. The long-tailed macaque (*Macaca fascicularis*) is a widespread non-human primate found across Southeast Asia, including Thailand, Vietnam, Indonesia, East Timor, Malaysia, and the Philippines [14]. In Thailand, it is protected under the Wildlife Conservation and Protection Act, B.E. 2535 (1992). Although earlier surveys reported its national distribution [15], recent data are lacking. Urban expansion and tourism may have altered macaque behavior [14]. These animals now live closer to people in temples, parks, and community forests. They often eat food given by humans and drink from water sources that may be contaminated. Fecal contamination of nearby water sources, particularly in areas with open drainage or shared water use, may serve as a route of transmission for intestinal parasites.

Several zoonotic GI parasites have been reported in macaques, such as *Strongyloides* spp., *Ascaris* spp., *Trichuris* spp., *Entamoeba histolytica*, and *Balantioides coli* [16,17,18,19,20]. These parasites can spread not only between macaques and humans, but also between macaques and domestic animals [21,22,23]. Northeastern Thailand is considered a high-risk area due to frequent overlap between human and macaque habitats [24]. Although some studies have examined GI parasites in macaques from this region (e.g., Udon Thani, Maha Sarakham, and Amnat Charoen Provinces) [17,18,25,26], the geographic coverage is limited.

In this study, we aimed to investigate the prevalence and species diversity of GI parasites in long-tailed macaques from four provinces (Loei, Khon Kaen, Bueng Kan, and Sisaket) in Northeast Thailand. The findings are intended to improve understanding of zoonotic risks at the human–wildlife interface and support the development of targeted One Health interventions in regions where humans and macaques cohabitate.

## 2. Materials and Methods

### 2.1. Study Design and Location

**Figure 1:**
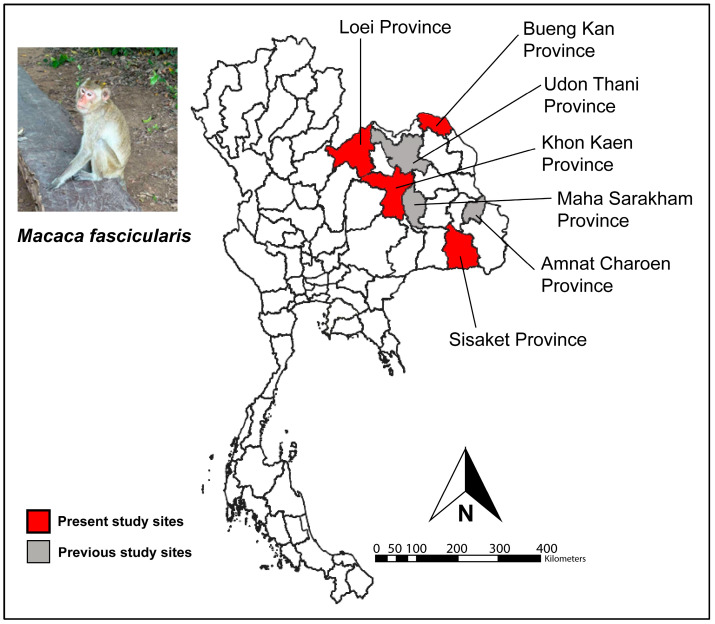
Map showing the locations of long-tailed macaque (*Macaca fascicularis*) study sites in Northeast Thailand. Red color indicates the provinces surveyed in the present study (Loei, Khon Kaen, Bueng Kan, and Sisaket). Sampling locations included Wat Tham Pha Mak Ho, Loei Province (*n* = 121); Wat Tham Pha Chor, Khon Kaen Province (*n* = 125); Ban Sang Community Forest, Bueng Kan Province (*n* = 105); and Ban Mueang Kan Monkey Park, Sisaket Province (*n* = 94). Gray color represents locations previously studied for gastrointestinal parasites in macaques, as previously reported [17,18,25,26]. The map was created by the authors using QGIS version 3.40. All other layers were produced by the authors and are copyright-free.

A cross-sectional study was conducted to investigate the prevalence of GI parasitic infections among long-tailed macaques (*M. fascicularis*) inhabiting natural environments in Northeast Thailand, across four provinces: Loei, Khon Kaen, Bueng Kan, and Sisaket (Figure 1). Field sampling was conducted from early April to mid-May 2025, and laboratory analysis was completed by late May 2025. The selected sites were Wat Tham Pha Mak Ho in Loei Province (approximately 300 macaques), Wat Tham Pha Chor in Khon Kaen Province (500 macaques), Ban Sang Community Forest in Bueng Kan Province (400 macaques), and Ban Mueang Kan Monkey Park in Sisaket Province (200 macaques). These locations were selected due to frequent human–macaque interactions, commonly occurring in temples, community forests, and public parks. Additionally, these locations also had not been included in earlier national surveys of free-ranging macaque populations. At all sites, long-tailed macaques forage on natural food sources such as leaves, fruits, and insects, but frequently rely on anthropogenic food, including waste and food provided by local residents, tourists, and religious practitioners. Habitat fragmentation and increased human activity have driven macaques closer to urban environments, resulting in high human–wildlife contact.

**Figure 2:**
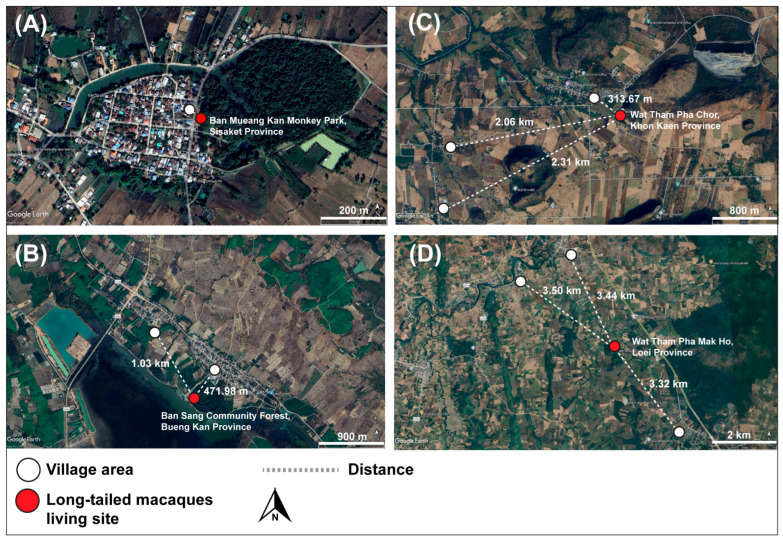
Satellite maps show the distance between long-tailed macaque habitats (red dots) and nearby village areas (white dots) in four provinces: (**A**) Sisaket; (**B**) Bueng Kan; (**C**) Khon Kaen; and (**D**) Loei. Dashed lines show the shortest path between macaques and people. Macaques in Sisaket and Bueng Kan live less than 500 m from villages. These short distances show high contact between humans and macaques. The map was created by the authors using Google Earth. All other layers were also produced by the authors and are copyright-free.

Figure 2 illustrates the spatial proximity between long-tailed macaque habitats and nearby human settlements in all four study locations. In some areas, such as Sisaket and Bueng Kan Provinces, macaque living sites were located within or at close distance (<500 m) to residential zones, suggesting a high degree of human–wildlife interface. The minimal spatial barriers between macaques and villagers underscore the potential for fecal–oral transmission of gastrointestinal parasites via contaminated environments.

### 2.2. Sample Size Calculation

Sample size was calculated using Wayne W. Daniel’s formula [27]. Assuming a parasite prevalence of 66% (*p* = 0.66) based on a previous study [26] with a 95% confidence level (z = 1.96) and a 5% margin of error (d = 0.05), the minimum sample size required was 345. An additional 10% dropout was added, yielding a final minimum sample size of 380.

### 2.3. Sample Collection

Fresh fecal samples were collected non-invasively from sites frequented by long-tailed macaques. To minimize the risk of environmental contamination, only the upper portion of each fecal sample, which had not come into direct contact with the ground, was collected. Sampling was performed along a single forward path without retracing steps to prevent duplicate collection from the same individual macaques. Only freshly excreted feces with intact morphology and visible moisture were collected. Each sample was placed in a clean fecal container, thoroughly mixed, and divided for analysis. One portion was used for agar plate culture (APC), while the remaining sample was preserved in 10% formalin for the formalin–ethyl acetate concentration technique (FECT). All samples were stored in transport boxes and delivered to the laboratory at the Faculty of Medicine, Mahasarakham University.

### 2.4. Parasitological Examination

Two parasitological diagnostic techniques (APC and FECT) were used for GI parasite detection. The APC is sensitive to *Strongyloides* sp. detection [28,29], while FECT is used to detect helminth eggs and/or larvae and protozoan cysts and/or trophozoite infections [30,31].

For the APC method, approximately 3 g of fresh fecal samples were placed in the center of a round nutrient agar plate (100 mm × 15 mm) at the field, then transferred to the Faculty of Medicine, Mahasarakham University. All fecal sample plates were incubated at room temperature for three to five days [32]. The cultural plates were examined daily under a stereomicroscope. Positive plates were washed and centrifuged. Parasites were stained for morphological identification under a light compound microscope. Positive plates were noted, while negative plates were re-examined on Days 4 and 5.

For the FECT method, two grams of fecal samples were filtered through double-layered surgical gauze and centrifuged at 2500 rpm for 5 min. The resulting sediment was processed with 7 mL of 10% formalin and 3 mL of ethyl acetate, shaken vigorously, and centrifuged again. Two drops of the final sediment were placed on glass slides, stained with 1% iodine, and examined under light microscopy at 100× and 400× magnification for the identification of helminth eggs and protozoan cysts. Two experienced parasitologists independently performed the examination.

### 2.5. Statistical Analysis

The prevalence of gastrointestinal parasitic infections is calculated by dividing the number of positive samples by the total number of samples collected and multiplying by one hundred. Chi-square tests were performed to compare prevalence rates between locations. A *p*-value < 0.05 was considered statistically significant. All analyses were performed using the STATA package version 10.1 (StataCorp LLC, College Station, TX, USA).

## 3. Results

### 3.1. Overall Prevalence of GI Infections

Table 1 presents the prevalence of gastrointestinal parasitic infections among free-ranging long-tailed macaques in four provinces of Northeast Thailand. Out of 445 fecal samples, 385 were positive for at least one gastrointestinal parasite, giving an overall prevalence of 86.52% (95% CI: 83.00–89.40). The highest prevalence of GI infections was found in Khon Kaen Province (91.20%), followed by Loei (90.91%), Bueng Kan (90.48%), and Sisaket (70.21%). The differences in prevalence among the provinces were statistically significant (χ^2^ = 26.31, *p* < 0.001).

Helminth infections were more common than protozoan infections, found in 67.19% and 56.40% of samples, respectively. Mixed infections involving both helminths and protozoa were observed in 37.30% of the cases, while 29.89% had only helminth infections and 19.33% had only protozoan infections. Loei, Khon Kaen, and Bueng Kan provinces exhibited high overall prevalence rates (around 90%), whereas Sisaket showed a comparatively lower prevalence at 70.21%. Mixed infections were most frequent in Loei (54.55%) and least common in Sisaket (20.21%).

### 3.2. Distribution of Protozoan Species

**Figure 3:**
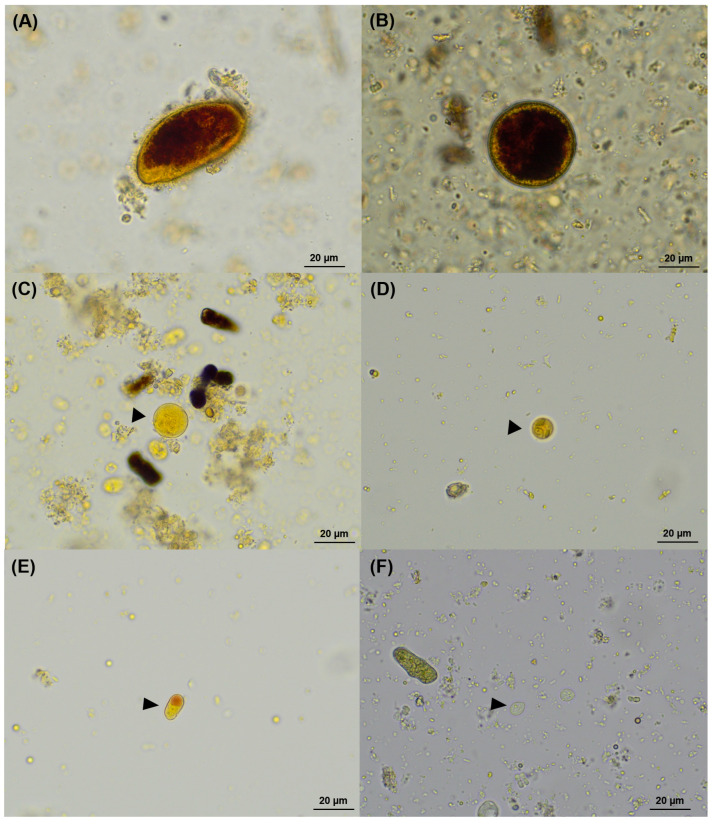
Microscopic images of protozoan parasites detected in fecal samples from long-tailed macaques. (**A**) *Balantioides coli*-like trophozoite; (**B**) *Balantioides coli*-like cyst; (**C**) *Entamoeba coli* cyst; (**D**) *Entamoeba histolytica*-like cyst; (**E**) *Iodamoeba bütschlii* cyst; (**F**) *Chilomastix mesnili* cyst. All images were captured under light microscopy at 400× magnification using iodine staining.

The most detected protozoan species was *Balantioides coli*-like (29.5%), followed by *Entamoeba histolytica*-like (28.8%) and *Iodamoeba bütschlii* (16.9%). Other protozoa included *Entamoeba coli* (12.4%), *Chilomastix mesnili* (2.7%), and *Blastocystis* sp. (0.2%). Province-specific data are shown in Table 2. Microscopic images of protozoan stages are presented in Figure 3.

### 3.3. Distribution of Helminth Species

**Figure 4:**
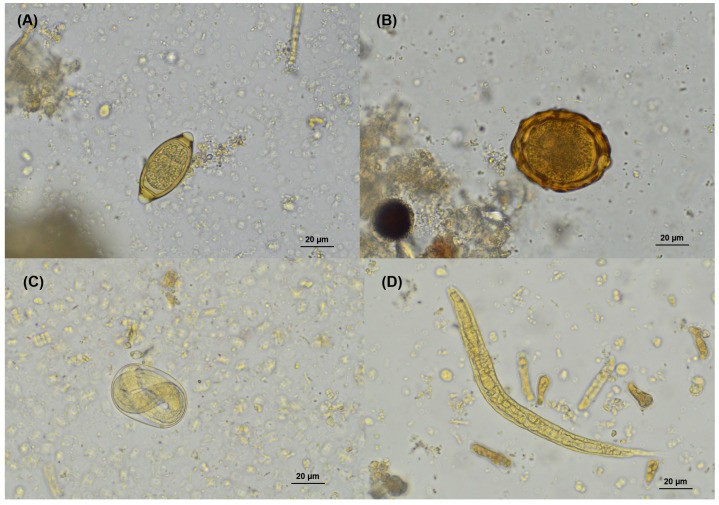
Microscopic images of helminth stages detected in fecal samples from long-tailed macaques. (**A**) *Trichuris* sp. egg; (**B**) *Ascaris* sp. fertilized egg; (**C**) *Strongyloides* sp. egg; (**D**) *Strongyloides* sp. rhabditiform larva. All images were captured under light microscopy at 400× magnification using iodine staining.

*Strongyloides* sp. was the most common helminth, found in 65.2% of the samples and detected in all four provinces. The highest prevalence was in Bueng Kan (79.0%) and the lowest was in Sisaket (40.4%). Other helminths included *Trichuris* sp. (347.2%) and *Ascaris* sp. (1.6%), with *Ascaris* sp. found only in Loei (Table 3). Microscopic images of helminth eggs and larvae are shown in Figure 4.

### 3.4. Diagnostic Comparison Between APC and FECT Methods for Strongyloidiasis

The APC method identified *Strongyloides* sp. in 62.0% of samples, while the FECT method detected it in only 41.4% of samples. The APC method was approximately 1.5 times more sensitive than FECT. Notably, 38.2% of samples tested positive for *Strongyloides* sp. using both methods, 23.8% were positive only by APC, and 3.2% were positive only by FECT (Table 4).

## 4. Discussion

This study found a high prevalence of GI parasitic infections (86.52%) in long-tailed macaques from four provinces in Northeast Thailand. Similar findings have been reported in other provinces of Thailand [26] and in countries such as Indonesia [33], Philippines [34], Laos [17], and Malaysia [35]. In the present study, we found that the differences in prevalence among the provinces were statistically significant. The highest prevalence of GI infections was found in Khon Kaen Province, followed by Loei, Bueng Kan, and Sisaket. These observed differences in prevalence among provinces may be attributed to environmental factors, macaque population density, and varying degrees of human–wildlife interface. Loei and Khon Kaen, where prevalence was highest, are known to have temple and park environments where macaques live in close proximity to humans and frequently receive food offerings. Such interactions may increase exposure to contaminated food and water sources, facilitating transmission of both protozoan and helminth parasites. Bueng Kan showed a particularly high prevalence of helminths, which could be linked to environmental conditions favorable for the development and survival of helminth eggs and larvae, such as moist soil and shared water access points. In contrast, Sisaket had the lowest overall prevalence and the lowest rate of mixed infections, possibly due to differences in macaque population density, limited human–wildlife interaction, or more dispersed ranging patterns, reducing opportunities for cross-contamination.

The most common parasite detected in this study was *Strongyloides* sp. This aligns with previous studies reporting a high prevalence of GI parasites in macaques in Kumphawapi Monkey Garden (Udon Thani Province) and Dong Ling Don Chao Pu Park (Amnat Charoen Province), Thailand [26]. In our study, APC was also used and may have improved detection sensitivity. Other parasites identified included helminths (*Trichuris* sp., *Ascaris* sp.) and protozoa (*B. coli*-like, *E. histolytica*-like, and *Blastocystis* sp.). Variation in the species diversity of GI parasites across regions may be influenced by host factors, geographic environmental differences, seasonal variation, and the diagnostic methods employed. However, our results, together with previous reports, support that long-tailed macaques in Asian countries carry a high burden of GI parasites [19,20,36].

Several GI parasites identified in this study are zoonotic and can infect humans [18,37]. They can cause mild digestive symptoms or serious disease [38]. *Strongyloides* sp. can enter the human body through skin contact with contaminated soil. Other parasites can be transmitted by eating or drinking contaminated food or water, or through contact with contaminated surfaces or vectors [17,38]. Since macaques often live near domestic animals, indirect transmission between species is also possible. Close contact between humans and macaques, especially in temples, parks, and tourist sites, increases the risk of disease transmission. Human behaviors such as feeding macaques, coupled with inadequate sanitation and shared water sources, facilitate parasite transmission. Therefore, GI parasites in macaques are not only an animal health problem but also a public health concern. Addressing this issue requires cooperation between different sectors.

The presence of multiple gastrointestinal parasite species and frequent mixed infections suggests that the environment may be heavily contaminated. Macaques may experience prolonged exposure to these parasites. Although molecular methods were not used in this study, the observed parasite forms warrant further investigation due to their medical significance. Previous molecular studies have identified *Strongyloides fuelleborni* as the predominant species in Southeast Asian macaques [17,20,39,40]. This species has also been reported to infect a human community having contact with long-tailed macaques in Udon Thani Province, Thailand—an area geographically adjacent to our study sites [41]. This human case highlights the zoonotic potential of macaque-associated *Strongyloides* and underscores the need for integrated surveillance in regions with high human–wildlife interaction. In the same geographic regions, previous studies have also identified *Trichuris trichiura* [16,25] and possibly *Trichuris ovis* in macaques [25]. In addition, a global meta-analysis found that zoonotic *Blastocystis* subtypes (ST1–ST8) in dogs and cats also occur in macaques [42]. *Blastocystis* sp. ST1–ST4 has also been commonly found in humans in Thailand [43,44]. It is important to note that *Balantidium coli* is now considered a synonym of *B. coli*. However, the genus name *Balantidium* refers to different species found in amphibians and fishes. *Balantioides coli* is the mammalian ciliate, as established by Chistyakova et al. [45], although morphologically similar species such as *Buxtonella* sp. have also been reported in *M. fascicularis* [46,47]. These organisms cannot be reliably distinguished using conventional light microscopy alone. The designation "*B. coli*-like" was used in this study based on morphology. Similarly, *E. histolytica*-like organisms are commonly found in this study. While some are pathogenic, others, such as *Entamoeba dispar*, are non-pathogenic but morphologically indistinguishable. Given the lack of molecular data and the taxonomic uncertainties, future studies should apply molecular techniques to confirm species identity and clarify transmission risks.

From a One Health perspective [48], our results show the shared risk of GI parasitic infections to humans and wildlife. Zoonotic parasites can spread through environmental contamination, especially in places where people and macaques live close together. The satellite maps in Figure 2 show that macaques live near human communities. In some areas, they live inside or next to villages. People feed them or walk through their areas. This close contact can lead to parasite transmission through soil, water, or shared space. These findings support the need for One Health planning in these areas. To reduce this risk, we recommend practical measures. These include signs to stop people from feeding macaques, improved waste management, and regular health checks for people and domestic animals in high-risk areas. Integrated surveillance systems should be developed. These systems should monitor macaque health, check for fecal contamination in the environment, and screen human communities. Early detection will help prevent disease outbreaks. Public health actions should focus on hotspots such as temples, parks, and forest edges. Health screening policy should include both macaques and people, especially children and other vulnerable groups.

This study has several limitations. Firstly, we used morphological techniques such as APC and FECT that are practical and widely used in field-based parasitological surveys, but they have limited sensitivity for detecting low-intensity infections and are often unable to distinguish morphologically similar or cryptic species. Incorporating molecular diagnostic methods, such as PCR or sequencing, in future studies would improve species-level identification and enhance the accuracy of epidemiological assessments. Secondly, the cross-sectional design, confined to a single season, limits inference on temporal trends. Additionally, host demographic data were not recorded, and behavioral or ecological factors potentially affecting the transmission risk of macaques were not assessed. Spatial analyses indicated close proximity between macaques and human settlements, but environmental sampling and transmission modeling were beyond the scope of this study. Thirdly, our sampling occurred during the hot and early raining season (April–May), which may influence parasite transmission dynamics. Seasonal changes in humidity and temperature are known to affect parasite life cycles. In addition, host factors such as age and sex may influence susceptibility to infection. Future studies should include multi-seasonal sampling and demographic stratification to better understand these effects. Despite these limitations, the findings of our study provide a valuable platform for evidence-based interventions. Moving forward, we recommend the development of community-based surveillance networks that integrate molecular diagnostics, environmental monitoring, and participatory risk communication. These systems can support early detection and targeted prevention in human–wildlife interfaces characterized by high parasitic burden.

## 5. Conclusions

This study highlights a high prevalence and diversity of gastrointestinal parasites in free-ranging long-tailed macaques in Northeast Thailand, with implications for zoonotic transmission. *Strongyloides* sp., *B. coli*-like, and *E. histolytica*-like organisms were commonly detected. Our findings underscore the need for integrated One Health strategies, including improved waste management in macaque-inhabited areas, targeted health education for communities living near wildlife, and routine parasite monitoring in both macaques and humans. Such approaches are critical to minimizing cross-species transmission and supporting sustainable wildlife–human coexistence.

## Figures and Tables

**Table 1:** Prevalence of gastrointestinal parasite infections among long-tailed macaques across four provinces in Northeast Thailand (*n* = 445).

Type of Infection	Loei (*n* = 121)	Khon Kaen (*n* = 125)	Bueng Kan (*n* = 105)	Sisaket (*n* = 94)	Total (*n* = 445)	χ^2^	*p*-Value *
	*n* (%)	*n* (%)	*n* (%)	*n* (%)	*n* (%)		
**No. Positive (%)**	110 (90.91)	114 (91.20)	95 (90.48)	66 (70.21)	385 (86.52)	26.31	<0.001
**95% CI**	84.45–94.85	84.93–95.02	83.35–94.74	60.32–78.52	83.00–89.40		
**Protozoa**	95 (78.51)	74 (59.20)	38 (36.19)	44 (46.81)	251 (56.40)		
**Helminth**	81 (66.94)	91 (72.80)	86 (81.90)	41 (43.62)	299 (67.19)		
**Mono Protozoa**	29 (23.97)	23 (18.40)	9 (8.57)	25 (26.60)	86 (19.33)		
**Mono Helminth**	15 (12.40)	40 (32.00)	56 (61.90)	22 (23.40)	133 (29.89)		
**Mixed Infection**	66 (54.55)	51 (40.80)	30 (28.57)	19 (20.21)	166 (37.30)		

Helminth: *Strongyloides* spp.; *Ascaris* spp.; *Trichuris* spp. infections; * Based on chi-square testing.

**Table 2:** Distribution of protozoan species among long-tailed macaques across four provinces in Northeast Thailand (*n* = 445).

Province	No. Examined	Species of Protozoa Identified					
		Number of Positive Samples (%)					
		*Balantioides coli*-like	*Iodamoeba bütschlii*	*Enthamoeba coli*	*Enthamoeba histiolytica* like	*Blastocytis* sp.	*Chilomastix mesnili*
**Loei**	121	71 (58.68)	41 (33.88)	12 (9.92)	43 (35.54)	0 (0.00)	8 (6.61)
**Khon Kaen**	125	33 (26.40)	3 (2.40)	20 (16.00)	51 (40.80)	1 (0.80)	4 (3.20)
**Bueng Kan**	105	13 (12.38)	7 (6.67)	1 (0.95)	17 (16.19)	0 (0.00)	0 (0.00)
**Sisaket**	94	21 (22.34)	24 (25.53)	22 (23.40)	17 (18.09)	0 (0.00)	0 (0.00)
**Total**	445	138 (31.01)	75 (16.85)	55 (12.36)	128 (28.76)	1 (0.22)	12 (2.70)

**Table 3:** Distribution of helminth species among long-tailed macaques across four provinces in Northeast Thailand (*n* = 445).

Province	No. Examined	Species of Helminth Identified		
		Number of Positive Samples (%)		
		*Strongyloides* sp.	*Trichuris* sp.	*Ascaris* sp.
**Loei**	121	78 (64.46)	1 (0.83)	7 (5.79)
**Khon Kaen**	125	91 (72.80)	1 (0.80)	0 (0.00)
**Bueng Kan**	105	83 (79.05)	12 (11.43)	0 (0.00)
**Sisaket**	94	38 (40.43)	0 (0.00)	0 (0.00)
**Total**	445	290 (65.17)	14 (3.15)	7 (1.57)

Helminth: *Strongyloides* sp.; *Ascaris* sp.; *Trichuris* sp. infections.

**Table 4:** Comparative efficacy of APC and FECT methods for *Strongyloides* sp. detection (*n* = 445).

Province	No. Examined	Prevalence of *Strongyloides* sp. Detected by APC and FECT (%)						
		Total of Positive (APC+ FECT)	APC Positive	FECT Positive	Both APC and FECT Positive (Similar Results)	Only APC Positive but Negative by FECT	Only FECT Positive but Negative by APC	Ratio (APC: FECT)
**Loei**	121	78 (64.46)	75 (61.98)	48 (39.67)	45 (37.19)	30 (24.79)	3 (2.48)	1.6
**Khon Kaen**	125	91 (72.80)	87 (69.60)	64 (51.20)	60 (48.00)	27 (21.60)	4 (3.20)	1.4
**Bueng Kan**	105	83 (79.05)	81 (77.14)	51 (48.57)	49 (46.67)	32 (30.48)	2 (1.90)	1.6
**Sisaket**	94	38 (40.43)	33 (35.11)	21 (22.34)	16 (17.02)	17 (18.09)	5 (5.32)	1.6
**Total**	445	290 (65.17)	276 (62.02)	184 (41.35)	170 (38.20)	106 (23.82)	14 (3.15)	1.5

## Data Availability

The data supporting this study are available with this article and can be further requested from the corresponding author.
